# The DOF Transcription Factor *SlDOF10* Regulates Vascular Tissue Formation During Ovary Development in Tomato

**DOI:** 10.3389/fpls.2019.00216

**Published:** 2019-02-26

**Authors:** Pilar Rojas-Gracia, Edelín Roque, Mónica Medina, María Jesús López-Martín, Luis A. Cañas, José Pío Beltrán, Concepción Gómez-Mena

**Affiliations:** Department of Plant Development and Hormone Action, Biology and Biotechnology of Reproductive Development, Instituto de Biología Molecular y Celular de Plantas, CSIC-UPV, Valencia, Spain

**Keywords:** tomato, parthenocarpy, DNA with one finger, development, vascular tissue

## Abstract

The formation of fruits is an important step in the life cycle of flowering plants. The process of fruit development is highly regulated and involves the interaction of a complex regulatory network of genes in both space and time. To identify regulatory genes involved in fruit initiation in tomato we analyzed the transcriptomic profile of ovaries from the parthenocarpic *PsEND1:barnase* transgenic line. This line was generated using the cytotoxic gene barnase targeted to the anthers with the *PsEND1* anther-specific promoter from pea. Among the differentially expressed genes we identified *SlDOF10*, a gene coding a DNA-binding with one finger (DOF) transcription factor which is activated in unpollinated ovaries of the parthenocarpic plants. *SlDOF10* is preferentially expressed in the vasculature of the cotyledons and young leaves and in the root tip. During floral development, expression is visible in the vascular tissue of the sepals, the flower pedicel and in the ovary connecting the placenta with the developing ovules. The induction of the gene was observed in response to exogenous gibberellins and auxins treatments. To evaluate the gene function during reproductive development, we have generated *SlDOF10* overexpressing and silencing stable transgenic lines. In particular, down-regulation of *SlDOF10* activity led to a decrease in the area occupied by individual vascular bundles in the flower pedicel. Associated with this phenotype we observed induction of parthenocarpic fruit set. In summary, expression and functional analyses revealed a role for *SlDOF10* gene in the development of the vascular tissue specifically during reproductive development highlighting the importance of this tissue in the process of fruit set.

## Introduction

The reproductive phase of angiosperms is characterized by the appearance of flowers and fruits. The flower is the reproductive organ of the plant and contains the male and female reproductive organs. The formation and development of the fruit is closely linked to the formation of the flower and under the control of both environmental and hormonal factors. Accordingly, flower and fruit development require the joint and coordinated action of a network of transcription factors (TFs) that act throughout the regulation of gene expression (Karlova et al., 2014). The establishment of distinct transcriptional domains is a fundamental mechanism for determining different cell fates within tissues and organs (Moreno-Risueno et al., 2012). TFs regulate gene expression by binding specific *cis*-regulatory elements in the promoter region of the target genes. In tomato, at least 998 putative TFs have identified from 62 different TF families that correspond to 2.87% of the estimated total number of genes (Guo et al., 2008; The Tomato Genome Consortium et al., 2012).

The DNA binding with one finger (DOF) proteins constitute a plant-specific family of TFs harboring a DNA-binding domain, which forms a single zinc-finger (Noguero et al., 2013). The highly conserved DOF domain is a region of 52 amino acid residues structured as a Cys2/Cys2 (C2/C2) zinc finger that recognizes a *cis*-regulatory element containing the common core sequence 5′-(T/A)AAAG-3′ (Yanagisawa, 2004). Besides the N-terminal conserved DNA-binding domain, these proteins contain a more variable C-terminal transcriptional regulation domain having diverse amino acid sequences (Yanagisawa, 2001; Gupta et al., 2015). DOF proteins are present across plant lineage, from green unicellular algae to higher angiosperms, and represent a unique class of TFs having bifunctional binding activities with both DNA and proteins (Gupta et al., 2015). The number of *DOF* genes is quite variable among different species that ranges from 9 genes identified in *Physcomitrella patens* to 36 and 54 DOF genes identified in Arabidopsis and maize, respectively (Gupta et al., 2015).

DNA-binding with one finger TFs play key role in a variety of biological processes during development and in response to environmental stimulus. They are often associated to plant specific processes such as light-responsiveness, tuber formation, seed development, seed germination, flowering, and plant hormone responses [reviewed by Noguero et al. (2013)]. The DOF proteins are also involved in general cellular activities such as cell cycle progression, cell expansion, metabolism regulation, and more. During plant development these proteins regulate the formation of a diverse number of structures including stomata guard cells (Negi et al., 2013), pollen (Peng et al., 2017), and the vascular system (Konishi and Yanagisawa, 2007; Guo et al., 2009; Gardiner et al., 2010).

In tomato, 34 DOF proteins have been identified distributed in 11 chromosomes and classified in 4 classes and 6 clusters (Cai et al., 2013). In addition to the highly conserved DOF domain, up to 25 conserved domains have been identified in this gene family. These additional domains result in a high divergence in the structure of the genes between the different groups or subgroups (Cai et al., 2013). Despite the importance of this gene family during plant growth, only a small number of members have been functionally characterized in tomato. A group of five tomato DOF genes, homologous to *Arabidopsis* Cycling DOF Factors (CDFs) are reported to be involved in the control of flowering time and abiotic stress responses (Corrales et al., 2014). More recently, the *TDDF1* gene was characterized and shown to be involved in circadian regulation and stress resistance (Ewas et al., 2017). Therefore, additional work is required to fully understand the role of *DOF* genes during tomato plant growth and development.

Tomato is a horticultural crop of major economic importance worldwide. The identification of regulatory genes involved in the control of fruit set will provide new molecular targets to implement breeding programs in these species. In this work we compared the transcriptome of ovaries from wild-type and parthenocarpic tomato plants (*PsEND1::barnase*) looking for differentially expressed TFs. In these plants parthenocarpic fruit development is triggered by early anther ablation. We selected the *SlDOF10* gene from the DOF family of TFs for extensive expression analyses and functional characterization. Our results support a role for *SlDOF10* gene in the development of the vascular tissue and specifically during reproductive development in tomato. We also discuss the role of the vascular system in the control of fruit set in this species.

## Materials and Methods

### Plant Material and Growth Conditions

Tomato plants (*Solanum lycopersicum* L.) from the Micro-Tom (MT) cultivar were used as the wild-type genotype. The transgenic line *PsEND1:barnase* MT TR1d (Roque et al., 2007) was used in the transcriptomic analyses. Plants were grown in pots with coconut fiber at 25–30°C (day) and 18–20°C (night) and were irrigated daily with Hoagland’s solution. Natural light was supplemented with Osram lamps (Powerstar HQI-BT, 400 W) to get a 16 h light photoperiod.

The treatment with IAA (2000 ng/ovary; Duchefa) and GA_3_ (2000 ng/ovary; Duchefa) was carried out to unpollinated ovaries, on the day equivalent to anthesis, in 10 μl of 5% ethanol, 0.1% Tween 20 solution (Serrani et al., 2008). Control ovaries were treated with the same volume of solvent solution. Samples were collected in pools 10, 30, 60, and 120 min after the treatment, frozen in liquid N_2_ and kept at -80°C until processed for expression analysis.

For fruit analyses the four first inflorescences of 10 independent plants were collected. To asses facultative parthenocarpy 12 flowers from each genotype were emasculated 2 days before anthesis. After 18 days ovaries were collected and weighed on a precision balance.

### Microarray Experiment Design

A comparative gene expression profiling was conducted using the microarray chip TOM2 (Cornell University, United States), a long oligonucleotide array representing 11862 tomato unigenes. Total RNA was isolated from tomato ovaries of wild-type (MT) and transgenic (*PsEND1:barnase* MT-TR1d) plants collected at 5 different time points during development. The selected floral stages were 6, 4, and 2-days-before anthesis (dba); at anthesis and 2-days-after anthesis (daa). The experiment was conducted with three biological replicates for each sample and a RNA reference sample obtained by pooling equivalent amounts of all RNA samples. After labeling, the reference sample was mixed with each individual sample to be used as a probe (30 hybridizations) using a dye swap approach. Microarray hybridization with labeled cDNA was performed using the protocols provided by the Tomato Functional Genomics Database (TFGD) at http://ted.bti.cornell.edu/cgi-bin/TFGD/array/TOM2_hybridization.cgi. The microarray slide was scanned for spot intensity using GenePix 4000B scanner (Molecular Devices) at 10 μm resolution. Genepix Pro software was used to quantify the spot intensity after subtracting the background, and optimization of the appropriate signal to noise ratio.

### Microarray Data Analysis

Data files were imported into Acuity 4.0 (Axon Instruments), and background-subtracted intensity was normalized by using the Lowess normalization method (Yang and Speed, 2002) using Acuity default values (smoothing filter, 0.4; iterations, 3; δ = 0.01). Finally, only spots with valid values in 80% hybridizations were considered for further analyses. To detect differentially expressed genes, a one-way analysis of variance (ANOVA) was performed to compare the mean Lowess-normalized values for a gene between experimental groups (parthenocarpic and wild type). A *P*-value cutoff of 0.05 was used to flag genes as being differentially expressed. Mean values of differential genes were calculated from each sample as log2 values. For the visual presentation of the results showing differential expression of the genes between wild-type and transgenic lines, as well as for Wilcoxon rank sum test calculation, MapMan software was used (Thimm et al., 2004).

### Quantitative RT-PCR

Total RNA was extracted using the RNeasy Plant Mini Kit (Qiagen, Hilden, Germany) according to the manufacturer’s instructions. One microgram of total RNA was used to synthesize first-strand cDNA, using the SuperScript First-Strand Synthesis System for RT-PCR (Invitrogen, Carlsbad, CA, United States). Quantitative RT-PCR (qRT-PCR) was carried out using the SYBR GREEN PCR Master Mix (Applied Biosystems, Carlsbad, CA, United States) in an ABI PRISM 7000 Sequence detection system (Applied Biosystems, Carlsbad, CA, United States) following the manufacturer’s recommendations. In a single experiment, each sample was assayed in triplicate. Expression levels were calculated relative to the constitutively expressed *SlACTINE8* gene (Martin-Trillo et al., 2011) using the ΔΔCt method. qRT-PCR data were obtained using three biological replicates. Primers were designed using Primer Express software from Applied Biosystems and are listed in Supplementary Table S1.

### *In situ* Hybridization

RNA *in situ* hybridization with digoxigenin-labeled probes was performed on 8 μM longitudinal paraffin sections of tomato seedlings and inflorescences as described previously (Gomez-Mena and Roque, 2018). The RNA antisense and sense probes were generated using the T7 polymerases, using a fragment of *SlDOF10* (positions 289–734 from the ATG codon) cloned in both orientations into the pGEM-T Easy vector (Promega).

### Histological Techniques

For histological studies, tissue was fixed and embedded in paraffin or resin (Technovit 7100; Kulzer, Wehrheim, Germany). Thin sections (1 μm) were stained with 0.05% toluidine blue in 0.1 M phosphate buffer at pH 6.8 (O’Brien et al., 1964). For whole-mount GUS detection, tissues were fixed for 10 min in ice-cold 90% acetone and GUS activity was revealed by incubation in 100 mM NaPO_4_ (pH 7.2), 2.5 mM 5-bromo-4-chloro-3-indolyl-ß-D-glucuronide, 0.5 mM K_3_Fe(CN)_6_, 0.5 m MK_4_Fe(CN)_6_ and 0.25% Triton X-100. Plant tissue was incubated at 37°C for 20 h. After staining, chlorophyll was cleared from the samples by dehydration through an ethanol series. For GUS detection in sectioned tissues, seedlings were first stained for GUS, followed by fixation and sectioning as for *in situ* hybridization. Digital images were processed (cropping, brightness, contrast, and color balance) with Adobe Photoshop (Adobe Systems) and analyzed quantitatively using Image J^1^. Whole-mount GUS images were obtained from 10 z-stack images corresponding to different focal planes.

### Subcellular Localization of SlDOF10 Protein by Transient Expression in *N. benthamiana*

The *SlDOF10* coding sequence was cloned via Gateway LR reaction into the destination vectors pEarleyGate101 and pEarleyGate 104 (Earley et al., 2006) to obtain N- and C-terminal fusions to the yellow fluorescent protein (YFP). The constructs were transformed into *Agrobacterium tumefaciens* C58 GV3850 and overnight cultures were diluted in infiltration buffer and used to infiltrate 4 week-old *Nicotiana benthamiana* leaves (Sparkes et al., 2006). Observations were performed on leave disks 48 h after infiltration under a confocal scanning microscopy (LSM 780, Zeiss).

### Plasmid Construction and Stable Plant Transformation

To make the GUS reporter fusion, approximately 2.5-kb of the 5′ promoter region of the *SlDOF10* gene was amplified using Advantage 2 Polymerase (Clontech) and oligonucleotides DOF10pro-For and DOF10pro-Rev, then cloned in the pCR8 vector (Invitrogen). Destination vector pKGWFS7,0 (VIB/Gent, Belgium) was used to generate the *SlDOFpro*::GUS construct. To make the *35S::SlDOF10* construct, *SlDOF10* cDNA was amplified with oligos SlDOF10-ORF-for and SlDOF10-ORF-rev and inserted into pK2GW7 binary vector (VIB/Gent, Belgium) using Gateway technology (Invitrogen) that placed the cDNA under the cauliflower mosaic virus 35S promoter. The *SlDOF10-RNAi* construct was generated using a 428 bp fragment of *SlDOF10* gene (positions 349–777 from the ATG codon), amplified using primers SlDOF10-RNAi-For and SlDOF10-RNAi-Rev and cloned into pK7GWIWG2(I) vector (VIB/Gent, Belgium). The fragment used in this construct is located outside of the conserved motifs of the protein (DOF domain and bipartite NLS signal). Primers are listed in Supplementary Table S1.

These three binary vectors were then introduced into *A. tumefaciens* LBA4404 by electroporation. The cotyledon co-cultivation method (Ellul et al., 2003) was used to transform wild-type tomato plants (cv. Micro-Tom). The transgenic plants were screened on antibiotic plates and transformants were transferred to soil for propagation.

### Transactivation Assay in *Nicotiana benthamiana* Leaves

A reporter plasmid was generated that consists in a fusion 2 consecutive DOF binding motif (TAAAG), the minimal TATA region of the 35S promoter and the Firefly *Luciferase* (*LUC*) gene. The two copies of the DOF *cis*-DNA element were produced by annealing the complementary single-stranded oligonucleotides 2 × DOF For and 2 × DOF Rev (Supplementary Table S1). In the same plasmid, the *Renilla* (*REN*) LUC under the control of the CaMV 35S promoter was used as control. The effector plasmid contains the complete *SlDOF10* cDNA driven by the CaMV 35S promoter. *A. tumefaciens* C58C1 (pMP90) was transformed by electroporation with the independent constructs.

Equivalent amounts of the LUC fusion plasmid and effector plasmid (in combination with the suppressor of gene silencing p19) were coinfiltrated in 4 week-old *N. benthamiana* leaves. After infiltration, plants were incubated at 22°C with 16 h photoperiod for 2 days before analysis. The luciferase activity was measured using the dual-luciferase reporter assay system (Promega, United States) according to the manufacturer’s instructions. Relative light units were measured on a GloMax 96 Microplate Luminometer (Promega). The relative luciferase activity was calculated as the ratio between the LUC and the control REN luciferase activity. Four biological repeats were measured for each sample in three independent experiments.

### Statistical Analyses

Statistical treatments of the data were made using the SPSS program, version 16.0 for windows, IBM. The analyses were made by Student *t*-test and one-way ANOVA for *p* < 0.05 followed by Tukey correction for multiple comparisons; (*P* < 0.05). Different letters above the data bars represent significant differences between treatments.

## Results

### Identification of *SlDOF10* Gene and Sequence Analysis

**Figure 1:**
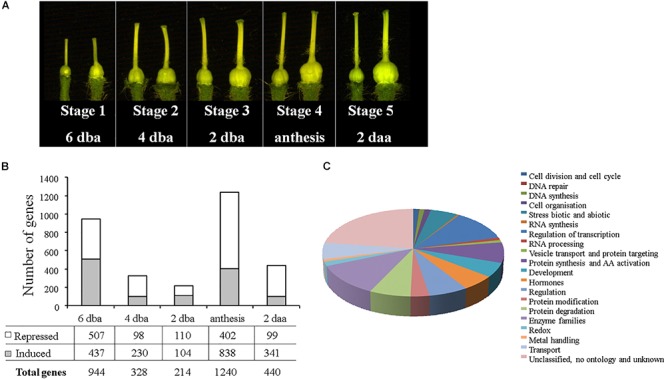
Identification of genes differentially expressed in ovaries from tomato parthenocarpic plants. **(A)** Dissected ovaries from the wild type (left) and parthenocarpic *PsEND1:barnase* plants (right) from the five developmental stages selected for the microarray experiments. **(B)** Number of genes differentially expressed in the five floral stages analyzed. **(C)** Functional categories of genes differentially expressed parthenocarpic ovaries during stage 1 according to MapMan software.

To identify regulatory genes that participate in the process of fruit set in tomato we looked for genes precociously activated in tomato parthenocarpic plants. We compared the transcriptome of ovaries form the parthenocarpic line *PsEND1*::*barnase* (Roque et al., 2007; Medina et al., 2013) and wild-type plants using the TOM2 oligo array (Figure 1). We compared the two samples over 5 independent time points that corresponded to 5 floral stages (6, 4, and 2 days before anthesis = dba; at anthesis and 2 days after anthesis = daa). The bigger number of changes in gene expression corresponded to stage 1 (6 dba) and stage 5 that corresponded to anthesis (Figure 1A). We have focused our study in the earliest floral stage that present significant gene modulation associated to precocious ovary growth in the parthenocarpic plants. Microarray analyses revealed 437 up-regulated and 507 down-regulated genes (Figure 1B and Supplementary Table S2) at this floral stage using a 2-fold threshold change. Genes were classified according to their annotated function and we selected a set of 89 unigenes were include in the functional category “Regulation of transcription” (Figure 1C and Supplementary Table S3).

Among the TFs differentially expressed we identify a unigene that presented a conserved domain characteristic of DOF TFs. Unigene SGN-U584226, corresponded to *SlDOF10* gene (*Solyc02g090310*) and was up-regulated in *PsEND1*::*barnase* ovaries 6 dba (Supplementary Figure S1). *SlDOF10* cDNA sequence was 1059 bp long and contained a 783 bp open reading frame flanked by 5′ untranslated (5′ UTR) and 3′ untranslated (3′ UTR) sequences of 97 and 176 pb, respectively. SlDOF10 protein contained a conserved N-terminal binding domain of 52 residues spanning a single C2/C2 zinc finger structure (DOF domain) and a bipartite nuclear localization signal (NLS) (Supplementary Figure S1) also described to be present in Arabidopsis DOF proteins (Krebs et al., 2010).

**Figure 2:**
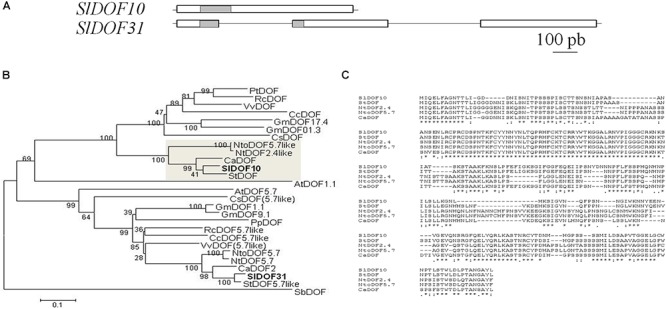
*SlDOF10* belong to a clade of DOF proteins conserved in the *Solanaceae* family. **(A)** Structure of *SlDOF10* and *SlDOF31* genes. White boxes indicate exons and gray boxes indicate DOF domains. **(B)** Phylogenetic tree for SlDOF10 protein and homologous proteins from several plant species. The SlDOF10 clade is highlighted by a gray square. *Sl, Solanum lycopersicum*; *St, Solanum tuberosum; Ca, Capsicum annuum; Nto, Nicotiana tomentosiformis; Nt, Nicotiana tabacum; Pt, Populus trichocarpa; Rc, Ricinus communis; Vv: Vitis vinifera; Md, Malus domestica; Gm, Glycine max; Cc, Citrus clementina; At, Arabidopsis thaliana; Sb, Sorghum bicolor*. **(C)** Protein alignment of DOF proteins from the SlDOF10 clade (marked by a gray box in panel a) showing stretches of conserved amino acids throughout the complete protein. Identical amino acids are marked by stars.

Genome-wide analysis of the tomato DOF family (34 members) revealed that gene family expansion originated after several duplication events where *SlDOF10* and *SlDOF31* are paralogs located on different chromosomes (Cai et al., 2013). However, *SlDOF10* and *SlDOF31* genes strongly differed in exon/intron structure in terms of intron number and exon length (Figure 2A) and presented low protein homology outside the DOF domain (Supplementary Figure S2). To analyze the duplication event of these paralogs in the context of time we inferred a phylogeny using a nucleotide dataset containing the two paralogs and several DOF homologs from a variety of species. The topology of the phylogenetic tree showed that SlDOF10 and SlDOF31 proteins placed in different clades, suggesting that the duplication resulting in these paralogs occurred early and prior to the speciation of the *Solanaceae* species included in the phylogenetic tree (Figure 2B). The homology among the proteins included in the SlDOF10 clade is not restricted to the DOF domain but extended to several stretches of amino acids throughout the whole protein (Figure 2C). Our data indicate a strong structural divergence of the two paralogs after duplication both at the DNA and protein level that might result in functional diversification.

### SlDOF10 Protein Is Located in the Nucleus and Shows Transcriptional Activity

**Figure 3:**
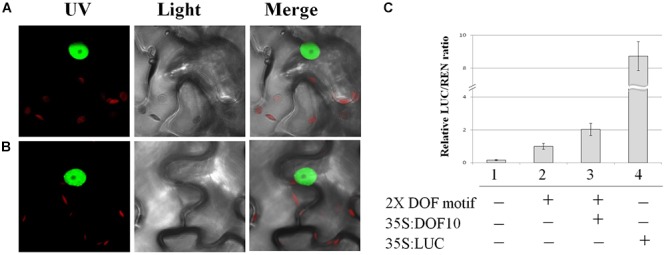
Subcellular localization and transactivation activity of SlDOF10 protein. **(A,B)** Nuclear localization of the YFP-SlDOF10 and SlDOF10-YFP fusion proteins. **(C)** Transient dual-LUC reporter assays showing SlDOF10 ability to activate transcription. A reporter construct with or without DOF binding sites (2 × DOF motif) was coexpressed with SlDOF10 protein and or the corresponding empty vector in the indicated combinations. 35S:LUC was used as a positive control.

SlDOF10 protein contains a highly conserved bipartite NLS characteristic of this family of proteins (Krebs et al., 2010). In order to determine the subcellular location of SlDOF10 we fused the YFP to either the C and N terminal part of the protein. YFP-tagged proteins were transiently expressed in *N. benthamiana* leaves and observed under the confocal microscope. The fusion protein localized exclusively to the nucleus of the epidermal cells (Figure 3A,B) consistent with a role for SlDOF10 as a transcription factor.

It has been reported that DOF proteins bind to the (T/A)AAAG core sequence motif found in many plant promoters (Mena et al., 2002; Yanagisawa, 2004). To test the transcriptional activation activity of SlDOF10 we performed transient transactivation assays in *N. benthamiana* leaves. The reporter constructs contains 2 consensus DOF-binding sequences and a minimal 35S promoter. The effector plasmid expressing the full-length NtSVP protein was also constructed (Figure 3C). When both plasmids were co-expressed the expression of the LUC reporter was significantly activated compared to negative controls (Figure 3C). These results support the idea that SlDOF10 has the ability to bind specific DNA sequences and activate transcription.

### *SlDOF10* Expression During Plant Development

**Figure 4:**
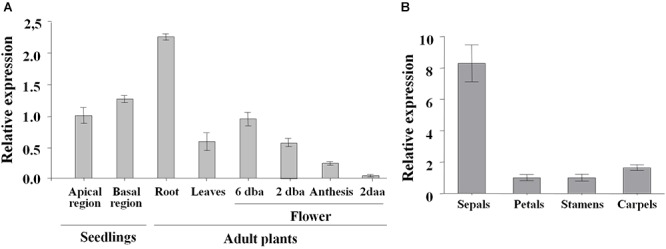
Expression pattern of *SlDOF10* during development in tomato plants. **(A)** Expression level of *SlDOF10* measured using qRT-PCR in seedlings (shoot apices and roots) and adult plants (roots, leaves and flowers). **(B)** Expression of *SlDOF10* in dissected floral organs from flowers at anthesis. dba, days before anthesis; daa, days after anthesis. Data were normalized to the expression of *SlACT8* and correspond to the mean (±SD) of three biological replicates.

In order to understand the function of *SlDOF10* gene during plant development we studied the expression patterns of the gene in different tissues from seedlings and adult plants. qRT-PCR analyses showed that the gene is expressed during both vegetative and reproductive development (Figure 4A). *SlDOF10* was expressed in 2 week-old seedling in the apical region containing the cotyledons, shoot and leaf primordia and in the basal region (hypocotyl and root) and in expanded true leaves and roots from adult plants. In the reproductive organs the expression of *SlDOF10* was detected at early stages of flower development (6 dba) and decreases during flower maturation (Figure 4A). In dissected floral organs from flowers at anthesis the expression was higher in sepals than in the other floral organs (Figure 4B). Among the analyzed tissues the higher levels of expression was observed in the roots from 1 month-old adult plants.

**Figure 5:**
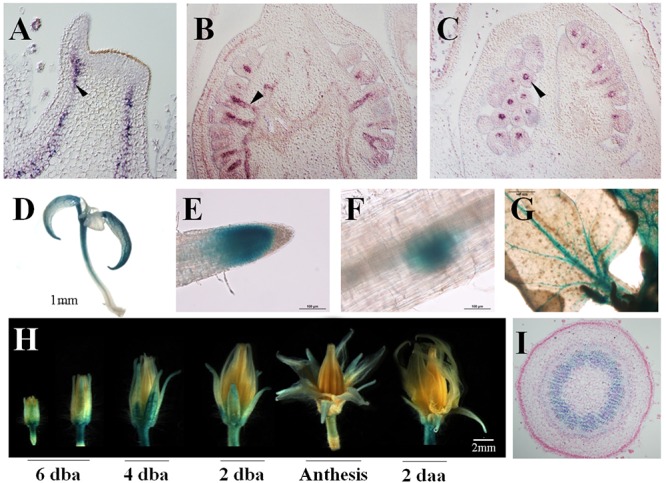
Expression pattern of *SlDOF10* during development in tomato plants. SlDOF10 expression detected by *in situ* hybridization in shoot apices **(A)** and ovaries **(B,C)**. *SlDOF10pro::GUS* expression in seedlings **(D)**, root apex **(E)**, secondary root primordia **(F)**, young leaves **(G)**, flowers **(H)** and a transversal section of the flower pedicel **(I)**.

The expression of the gene at tissular level was analyzed performing *in situ* hybridizations. *SlDOF10* mRNA was visible in apical shoots from 2 week-old seedlings and ovaries from 2 dba flowers, specifically localized in the vascular tissue (Figure 5A). In ovaries, the signal was observed in the vascular tissues of the funiculus and the placenta (Figure 5B,C). We also generated reporter lines by fusing 2.4 Kb of the *SlDOF10* promoter to the GUS reporter gene and transformed tomato plants. Consistent with the *SlDOF10* pattern detected by *in situ* hybridization, *SlDOF10pro*::*GUS* expression in tomato plants was observed also in vascular tissue from seedling and adult plants (Figure 5D–I). *SlDOF10pro*::*GUS* seedlings showed expression of the GUS reporter in the vasculature of cotyledons, hypocotyls, root tips and lateral root primordia (Figure 5D,F). Fragment of leaf pedicels and stem were analyzed and showed no expression of the gene in the vascular tissue. However, we detected rapid activation of *SlDOF10pro*::*GUS* expression near wound sites although further analyses are required to stablish the wound-induced expression of the gene (Supplementary Figure S3). During floral development, expression accumulated in the receptacle, the pedicel and in the vascular tissue of sepals (Figure 5H). Transversal sections of *SlDOF10pro*::*GUS* flower pedicels showed expression activity in the vascular ring (Figure 5I). These results suggest that *SlDOF10* could have a transcriptional regulatory role on the formation of vascular tissues during reproductive development in tomato.

To evaluate whether the 2.4 kb fragment from *SlDOF10* promoter can be used as a tissue-specific marker for vascular tissues, we transformed *Arabidopsis thaliana* with the *SlDOF10pro*::*GUS* construct. In seedlings from the transgenic plants, *GUS* expression was observed in the vascular tissues of cotyledons, true leaves and primary roots (Supplementary Figure S4). During reproductive development GUS staining was visible in the vascular tissues of all floral organs (Supplementary Figure S2). As reported in tomato plants, in the ovary GUS staining is observed in the placenta and the vascular tissue of the funiculus (Supplementary Figure S4E). This pattern of expression was maintained in the mature fruit (Supplementary Figure S4G). These results indicate that the 2.4 Kb fragment form the *SlDOF10* promoter used in the construct contains *cis*-regulatory elements that are conserved across tomato and Arabidopsis species. Moreover, the promoter from the *SlDOF10* gene could be used as a vascular-tissue-specific promoter for additional studies.

### Functional Analysis of *SlDOF10* Gene

To elucidate the function of *SlDOF10* during plant development transgenic tomato plants with reduced levels of the gene (*SlDOF10*-RNAi) were generated. Additionally, as a complementary strategy gain-of-function lines (*35S:SlDOF10*) were also generated (see Materials and Methods for details of the constructs). The expression of the targeted gene was analyzed in the T0 RNAi lines (14) and 3 of the lines showed a reduction of 80% in the expression level of *SlDOF10* (Supplementary Figure S5A). Four independent T0 *35S:SlDOF10* plants were generated with increased *SlDOF10* transcript level that range from two- to fivefold (Supplementary Figure S5B). Vegetative growth was not altered in the overexpressing or RNAi transgenic lines as expected by the absence of expression in the plant stems and leaf pedicels (Supplementary Figure S3). These plants were able to produce flowers and fruits. Two RNAi lines (L29 and L31) and the overexpression line with the higher level of expression (L16) were selected for further characterization in the T2 generation.

**Figure 6:**
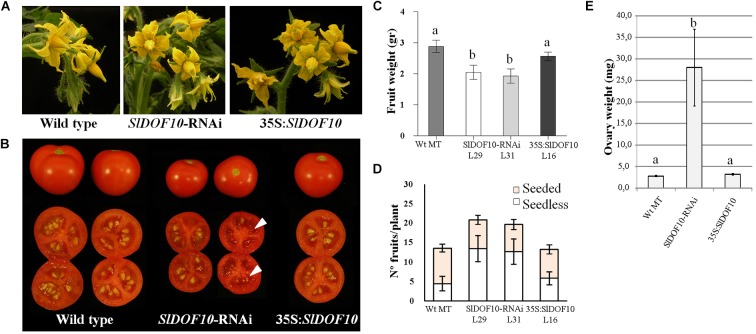
Effect of *SlDOF10* silencing or overexpression on the reproductive development of tomato plants. **(A)** Phenotype of flowers. **(B)** Phenotype of the fruits. *SlDOF10*-RNAi fruits occasionally showed presence of aborted seeds or pseudo embryos (arrows). **(C)** Fruit weight. **(D)** Number of seeded and seedless fruits. **(E)** Ovary growth after flower emasculation. Values are means of twelve ovaries ± SE (*n* = 12). Different letters above the data bars represent significant differences between treatments [one-way analysis of variance (ANOVA) followed by Tukey correction for multiple comparisons; *P* < 0.05].

Mild to severe defects were observed in the flowers of the RNAi lines that consisted in the incomplete fusion of the staminal cone (Figure 6A). These defects were shown by 45% of the flowers being only 16% severe defects on anther fusion. The overexpression line showed a greater proportion of affected flower (56%) and also higher rate of severe defects (27%). Despite these defects on stamen formation, overexpressing plants did not show alterations in the size of the fruits, the number of seeds or the formation of parthenocarpic fruits (Figure 6B,C). On the contrary, *SlDOF10*-RNAi lines showed smaller fruits than the wild type and a high number of seedless fruits (Figure 6B,C). In tomato, a relationship between fruit weight/size and seed content within a variety has been reported (Pet and Garretsen, 1983). Accordingly, the fruits from the *SlDOF10*-RNAi lines contained a reduced number of seeds and the occasional presence of pseudo-embryos (Figure 6B). However, histological sections of anthesis flowers showed that ovule development was not affected in the RNAi lines (Supplementary Figure S6). On the other hand results from Figure 6D showed that MT plants (the wild type genotype) has a natural tendency to produce seedless fruits under our growing conditions. This tendency is maintained in the *35S:DOF10* line and greatly enhanced in the *SlDOF10*-RNAi lines. Therefore, facultative parthenocarpy was evaluated in these plants by emasculation of unpollinated flowers. The ovaries form wild-type and overexpressing lines arrested growth whereas all the ovaries from the RNAi lines continued growing in the absence of pollination (Figure 6C). In the RNAi lines the weight of the ovaries (measured 18 days after emasculation) ranged from 3 to 33 times higher than the average ovary weight of the emasculated wild-type ovaries. This experiment suggests that silencing of *SlDOF10* gene promotes the autonomous growth of the ovary in the absence of pollination and fertilization.

**Figure 7:**
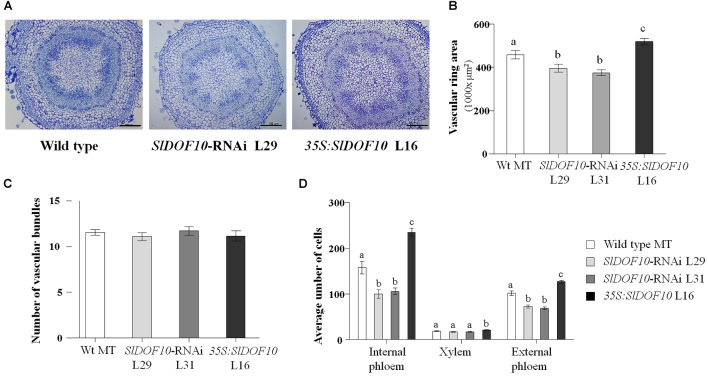
Transversal section of flower pedicels from flowers at anthesis from *SlDOF10*-RNAi, *35S:SlDOF10* transgenic plants and the wild type (MT). **(A)** Resin-embedded section of flower pedicels showing the vascular ring. **(B)** Quantification of the vascular ring area. **(C)** Quantification of the number of vascular bundles. **(D)** Cell number (xylem and phloem) in single vascular bundles. Scale bars are 100 μm. Different letters above the data bars represent significant differences at *P* < 0.05.

According to the expression analyses, *SlDOF10* transcript is located in the vascular tissue of the ovary (Figure 5B,C,I) that connects the ovules with the placental tissue and the flower pedicel. We then analyzed possible changes in the ovary vascular tissue development caused by altered function of the *SlDOF10* gene. We performed histological section of flower pedicels form the overexpressing and silenced transgenic lines. The pedicel is the nearest tissue connected with the ovary and the use of histological sections allows morphological studies of the vascular system in a two-dimensional distribution. Cross section of tomato pedicels showed a vascular ring with 10–12 vascular bundles. The arrangement of the vascular bundles is bicollateral, where xylem is lined with phloem on both its inner and outer faces (Figure 7A). Cross sections of flower pedicels showed that the area of the vascular ring was smaller in the *SlDOF10*-RNAi lines and bigger in the overexpressing lines when compared to the control (Figure 7B). However, these differences in the size of the vascular ring were not the result of changes in the number of vascular bundles (Figure 7C). Looking at the individual vascular bundles we observed alterations in their total area and also in the number of cells from the xylem and phloem. In fact, cell quantification showed higher number of cell in *35S:SlDOF10* and the opposite effect in the *SlDOF10*-RNAi line (Figure 7D). These results suggest a regulatory role for the SlDOF10 protein in the control of cell proliferation during the development of the vascular tissue in the flower.

### *In silico* Analysis of *Cis*-Acting Regulatory Elements in *SlDOF10* 5′ Regulatory Region

We scanned the 5′ regulatory region (2463 bp) used in the p*SlDOF10*::*GUS* construct for the presence of putative *cis*-acting regulatory elements registered in Plant CARE (Lescot et al., 2002) and PLACE (Higo et al., 1999). Several functional significant *cis*-acting regulatory elements associated with different processes in plant development were identified upstream of the *SlDOF10* gene. The names of the identified putative *cis*-acting elements and their predicted functions are tabulated in Supplementary Table S4. Among them we identified at least twenty *cis*-acting regulatory element involved in light responsiveness element (GAG-motif and G-box). Also, the region contains sequences involved in different stress response. Besides, we identify *cis*-acting regulatory elements involved in hormones responsiveness including cytokinins, salicylic acid, jasmonic acid, gibberellin, and auxin response (Supplementary Table S4).

**Figure 8:**
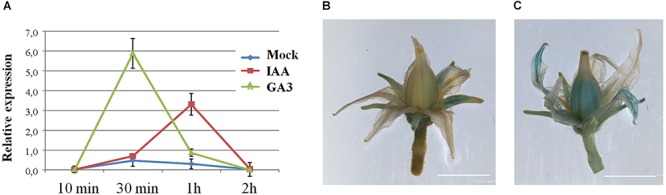
*SlDOF10* in upregulated by hormone treatments. **(A)** Relative expression of the *SlDOF10* gene was determined by qRT-PCR for floral apices treated with solvent (mock), IAA or GA_3_ and collected at different time points. **(B)** Expression of *SlDOF10* in tomato *SlDOF10pro::GUS* flowers of control plants (mock) and **(C)** after 1 h of treatment with IAA. Scales bars are: 5 mm.

We have paid special attention to *cis*-acting auxin regulatory elements because fruit set and development processes are initiated by auxin-induced changes in gene expression and followed by gibberellin (Serrani et al., 2008). Therefore, we subsequently treated tomato ovaries with auxin (IAA) and gibberellins (GA_3_) and examined the expression levels of *SlDOF10* by qRT-PCR. The results showed that auxin treatments rapidly activated *SlDOF10* expression after 30 min of the treatment, with maxima expression 1 h after auxin application (Figure 8A). Gibberellin treatment induces the rapid and strong activation of the gene after 30 min and then declined (Figure 8A). In addition we tested the expression of a reporter gene driven by 2.5-kb of the 5′ promoter region of the *SlDOF10* gene. A single exogenous treatment of flowers with auxin was sufficient to induce strong GUS expression in *SlDOF10pro*::*GUS* plants (Figure 8B,C) especially in the vascular system of sepals and in the stamens. Taken together these results suggest that *SlDOF10* gene could be regulated by gibberellins and auxins during reproductive development.

## Discussion

We have characterized *SlDOF10* gene, coding the first tomato DOF transcription factor known to be involved in the regulation of plant development. Our results revealed that SlDOF10 controls the formation of the vascular tissue during reproductive development. Several DOF proteins have been reported to regulate vascular system development in Arabidopsis. Indeed, in Arabidopsis half of the identified DOF TFs have been found expressed in the vascular tissues (Le Hir and Bellini, 2013). Most of the DOF genes characterized so far are required during the vegetative growth phase. Both *AtDof5.8* and *AtDof2.4* promoters become sequentially activated at early but distinct stages of procambium formation in leaf primordia. However, *AtDOF5.8* is also activated during the development of flower buds, in developing stamens at the early developmental stage and in carpels at the later developmental stages (Konishi and Yanagisawa, 2007). Despite the similarities between the pattern of expression of *SlDOF10* and *AtDOF5*.8 genes during flower development, phylogenetic analyses showed that they belong to separate clusters (Cai et al., 2013). Three additional members of the Arabidopsis DOF family (*DOF2.1*, *DOF4.6*, and *DOF5.3*) were also activated at early stages of vascular strand formation in the leaf (Gardiner et al., 2010). Experimental manipulation of leaf vascular patterning correlated with changes in the expression of these genes, suggesting that DOF expression identifies characteristic steps in vein ontogeny (Gardiner et al., 2010). The role of these genes during reproductive development was not investigated. Additional DOF genes have been identified using genetic approaches. *Dof5.6/HCA2* (*HIGH CAMBIAL ACTIVITY 2*) encodes a DOF protein with an EAR-motif associated with transcription repression. The *Dof5.6/HCA2* transcript was ubiquitously expressed in all the plant organs and *hca2* mutants showed pleiotropic effects on plant morphology. Interestingly, although the flowers of *hca2* mutants were normal, the *hca2* siliques were shorter and contained fewer seeds per silique (Guo et al., 2009). Similarly, down-regulation of *SlDOF10* activity affected fruit set and seed development (Figure 6).

Several genes from the DOF family have been identified to be specifically involved in seed development, including *DAG1*/*AtDOF3.7* (Papi et al., 2000; Gualberti et al., 2002), *AtDof6* (Rueda-Romero et al., 2012), and *DAG2* (Gualberti et al., 2002). In particular homozygous *dag1* plants showed twisted siliques with a reduced number of seeds that do not develop dormancy and germinate in the absence of light (Papi et al., 2000). This phenotype correlates with the expression pattern of the *DAG1 (AtDOF3.7)* gene that was observed in the gynoecium, specifically localized in the vascular tissue and the funiculus that connects the placenta to the ovule (Papi et al., 2000). This expression pattern is similar to the expression of *SlDOF10* in tomato ovaries (Figure 5B) and the pattern shown in *DAG2:GUS* lines (Gualberti et al., 2002) suggesting a common function for these genes. However, in the case of tomato, additional experiments are required to evaluate a possible role for *SlDOF10* during seed germination and dormancy. Taken together, the results from Arabidopsis and tomato suggest an important and conserved role for this subset of DOF genes during reproductive development and in particular in the formation of flowers and seeds.

Phylogenetic analyses of DOF family in Arabidopsis and rice revealed four major clusters and nine subfamilies of orthologous genes of subfamilies named A, B1, B2, C1, C2, C3, D1, D2, and D3 (Lijavetzky et al., 2003). The tomato, Arabidopsis and rice DOF families contain a similar number of members (34, 35, and 30, respectively) and similar phylogenetic relationships (Cai et al., 2013) suggesting that they may have evolved conservatively. In the tomato family segmental duplication is predominant for DOF gene evolution although tandem duplication is also involved giving rise to ten pairs of paralogous genes (Cai et al., 2013). *SlDOF31* gene was recognized as the putative paralog of SlDOF10 that probably resulted from ancient whole-genome duplication (Song et al., 2012). These two genes are located in two different chromosomes and showed important differences in the structure of the genes and the size and sequence of the protein (Figure 2 and Supplementary Figure S2). This strong structural divergence of the two paralogs and the non-overlapping expression patterns suggests that after duplication, functional diversification might occur. On the other hand, our results showed that *SlDOF10* is expressed in vegetative and reproductive tissues during plant development (Figure 4, 5). However, *SlDOF10-RNAi* plants did not show obvious defects during vegetative development possibly due to genetic or functional redundancy. In this regard, previous expression analyses of tomato DOF genes showed that *SlDof1*, *SlDof29*, *SlDof10*, and *SlDof32* have similar expression patterns (Cai et al., 2013) implying possible redundant functions. Nowadays, the lack of functional analyses of most of these genes does not permit to evaluate the genetic interactions among them.

*SlDOF10* gene encodes a protein of 260 amino acids with a well conserved DOF domain (Supplementary Figure S1). The protein was localized in the nuclei and showed transcriptional activity supporting its function as a transcription factor. Remarkably, SlDOF10 is the only protein from the tomato family where no additional conserved motifs were identified (Cai et al., 2013). Phylogenetic analyses using homologous proteins from different species showed that SlDOF10 cluster together with members of the *Solanaceae* family forming a small protein clade (Figure 2) being AtDOF1.1 the closest Arabidopsis homolog. *AtDof1.1* (*OBP2*) is part of a regulatory network controlling glucosinolate biosynthesis in Arabidopsis (Skirycz et al., 2006). Interestingly, *OBP2* expression was observed in the vascular tissue and stimulated by wounding and MeJA. Hormonal regulation of DOF expression was also reported in barley and rice in response to gibberellins (Mena et al., 2002; Washio, 2003) and in tobacco in response to auxin (Baumann et al., 1999). Our data indicate that *SlDOF10* expression is transcriptionally regulated by auxins and gibberellins during reproductive development, key regulatory elements on fruit set initiation (Goetz et al., 2006; Serrani et al., 2008). Moreover *SlDOF10* shows an overlapping expression pattern with the auxin response factors *ARF8* and *SlARF7* within the ovary (Goetz et al., 2006). A recent study using laser-capture microdissection and high-throughput RNA sequencing reported a comprehensive tissue-specific transcriptomic analysis during early tomato fruit development (Pattison et al., 2015). Interestingly, ten members of the C2C2-DOF family of TFs, including *SlDOF10*, form a coexpression cluster with several auxin related genes including the auxin-efflux carriers *PIN-FORMED1* (*PIN1*) and *PIN7*, two AUX/IAA family genes (*IAA13* and *IAA17*) involved in auxin signal transduction, and a GH3 family gene involved in auxin conjugation (Pattison et al., 2015). A transcriptional association between C2C2-DOF TFs and their potential target genes involved in auxin transport and signaling has been suggested (Pattison et al., 2015). Taken together, the data suggest that transcriptional regulation of *SlDOF10* and gene function largely depends on the hormone dynamics during tomato reproductive development.

Polar auxin transport controls multiple developmental processes in plants, including the formation of vascular tissue (Gälweiler et al., 1998). During tomato fruit development, the application of auxin transport inhibitors that block export of auxins from the ovary stimulates parthenocarpic fruit set (Serrani et al., 2010). In addition the down-regulation of the auxin efflux carrier *SlPIN4* leads to parthenocarpic fruit growth (Mounet et al., 2012). Also *SlPIN1* has been shown to plays central roles in leaf initiation and fruit development promoting the basipetal auxin efflux from the ovary to the flower pedicel (Shi et al., 2017). *SlDOF10* down-regulation reduced vascular tissue development in the flower pedicel (Figure 7) and induced parthenocarpic fruit growth. Precocious ovary growth could be the consequence of reduced polar auxin transport from the ovary leading to changes in the local distribution of hormones. Although additional experiments are needed to confirm this hypothesis, the functional analyses of *SlDOF10* gene highlight the importance of the vascular tissue in the process of fruit set.

In tomato, further work is needed to investigate the function of the DOF genes family during plant development. However, the functional characterization of *SlDOF10*, the first tomato DOF gene involved in vascular tissue formation, provides insight on the role of this family of TFs during reproductive plant development.

## Data Availability

All datasets generated for this study are included in the manuscript and/or the Supplementary Files.

## Author Contributions

CG-M and PR-G conceived and performed the experiments and analyzed the data. PR-G, ER, MM, and ML-M performed the experiments and analyzed the data. CG-M, JB, and LC wrote the grant that funded this work. CG-M, ER, JB, and LC wrote and reviewed and edited the manuscript.

## Conflict of Interest Statement

The authors declare that the research was conducted in the absence of any commercial or financial relationships that could be construed as a potential conflict of interest.

## Footnotes

1 http://rsb.info.nih.gov/ij/

## Supplementary Material

Figure S1 Gene sequence and conserved protein domains of the *SlDOF10 gene*. **(A)** Coding sequence and predicted protein sequence. Start and Stop codons are boxed. **(B)** Schematic representation of the SlDOF10 protein. The conserved amino acid of the DOF domain and the bipartite nuclear localization signal (NLS) are highlighted in red and blue, respectively.

Figure S2 Protein alignment of the putative paralogs SlDOF10 and SlDOF31. The position of the DOF domain and NLS is underlined.

Figure S3 Histochemical GUS staining of stems and leaf pedicels of *SlDOF10pro::GUS* transgenic tomato plants. **(A)** Leaf pedicel and **(B)** stem. Scales bars are: 2 mm.

Figure S4 Histochemical GUS staining of transgenic Arabidopsis carrying the GUS-coding region fused to *SlDOF10* promoter. *SlDOF10pro::GUS* expression in the vascular tissue of seedlings **(A)**, roots **(B)**, secondary roots **(C)**, and flowers **(D)**. Dissected flower showing GUS staining at the vascular tissue from the funiculus **(E)**. Mature fruit showing GUS staining in the apical and basal region and the margin of the valve **(F)**. Detail from mature fruit showing blue staining in the funiculus (Fu) and vascular tissue from the fruit **(G)**. Scale bars are: 1 mm.

Figure S5 Expression level of *SlDOF10 gene* in the RNAi and overexpressing lines. **(A)** Relative expression of *SlDOF10* in the *SlDOF10*-RNAi lines measured by qRT-PCR. **(B)** Relative expression of *SlDOF10* in the 355S:RNAi lines measured by qRT-PCR. The asterisks denote a significant difference between the transgenic lines and the wild type at *p* < 0.05.

Figure S6 Histological sections of ovules from flowers in anthesis. **(A)** Wild type ovules (Micro-Tom cv.). **(B)** *SlDOF10*-RNAi plants. **(C)** 35S: *SlDOF10* plants.

TABLE S1 Primers used in this work.

TABLE S2 Genes differentially expressed in the ovaries of the parthenocarpic line *PsEND1::barnase* at 6 days before anthesis (dba).

TABLE S3 Differentially regulated genes from the functional category “regulation of transcription.”

TABLE S4 Predicted *cis*-acting elements in SlDOF10 promoter region identified using PlantCARE and PLACE.
