# Supplementary figures and images for: The DOF Transcription Factor SlDOF10 Regulates Vascular Tissue Formation During Ovary Development in Tomato
Source: Front Plant Sci. 2019 Feb 26;10:216. doi: 10.3389/fpls.2019.00216 (PMC6399211; doi:10.3389/fpls.2019.00216)

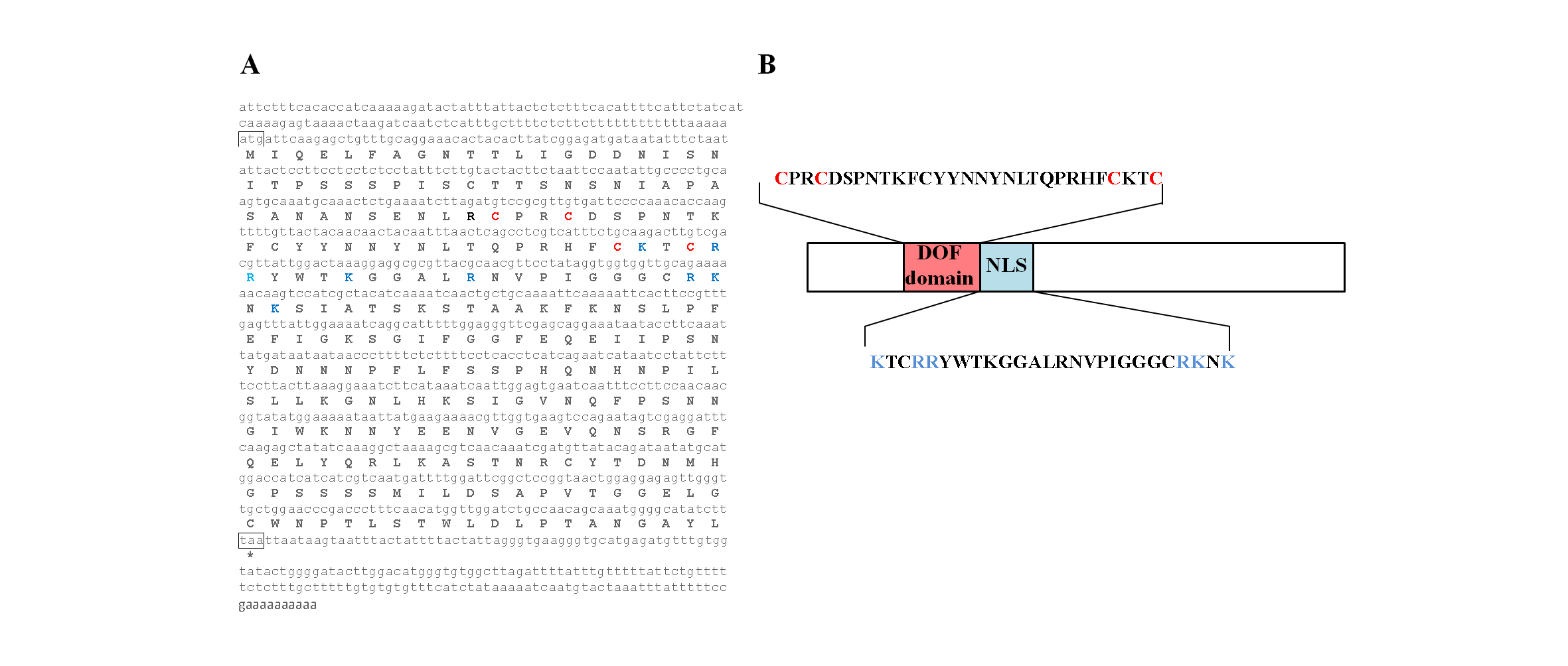

Supplement: Figure S1 — Gene sequence and conserved protein domains of the SlDOF10 gene. (A) Coding sequence and predicted protein sequence. Start and Stop codons are boxed. (B) Schematic representation of the SlDOF10 protein. The conserved amino acid of the DOF domain and the bipartite nuclear localization signal (NLS) are highlighted in red and blue, respectively. [file Image_1.JPEG]

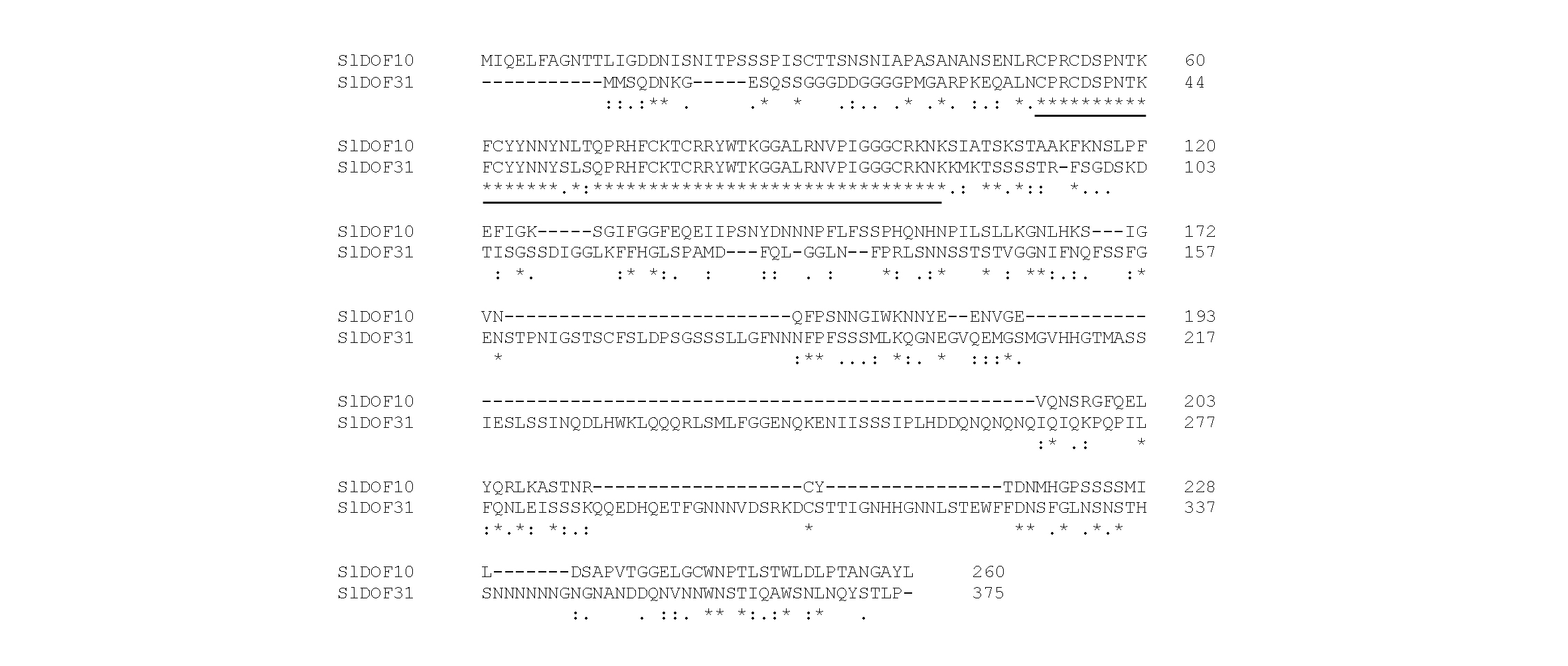

Supplement: Figure S2 — Protein alignment of the putative paralogs SlDOF10 and SlDOF31. The position of the DOF domain and NLS is underlined. [file Image_2.JPEG]

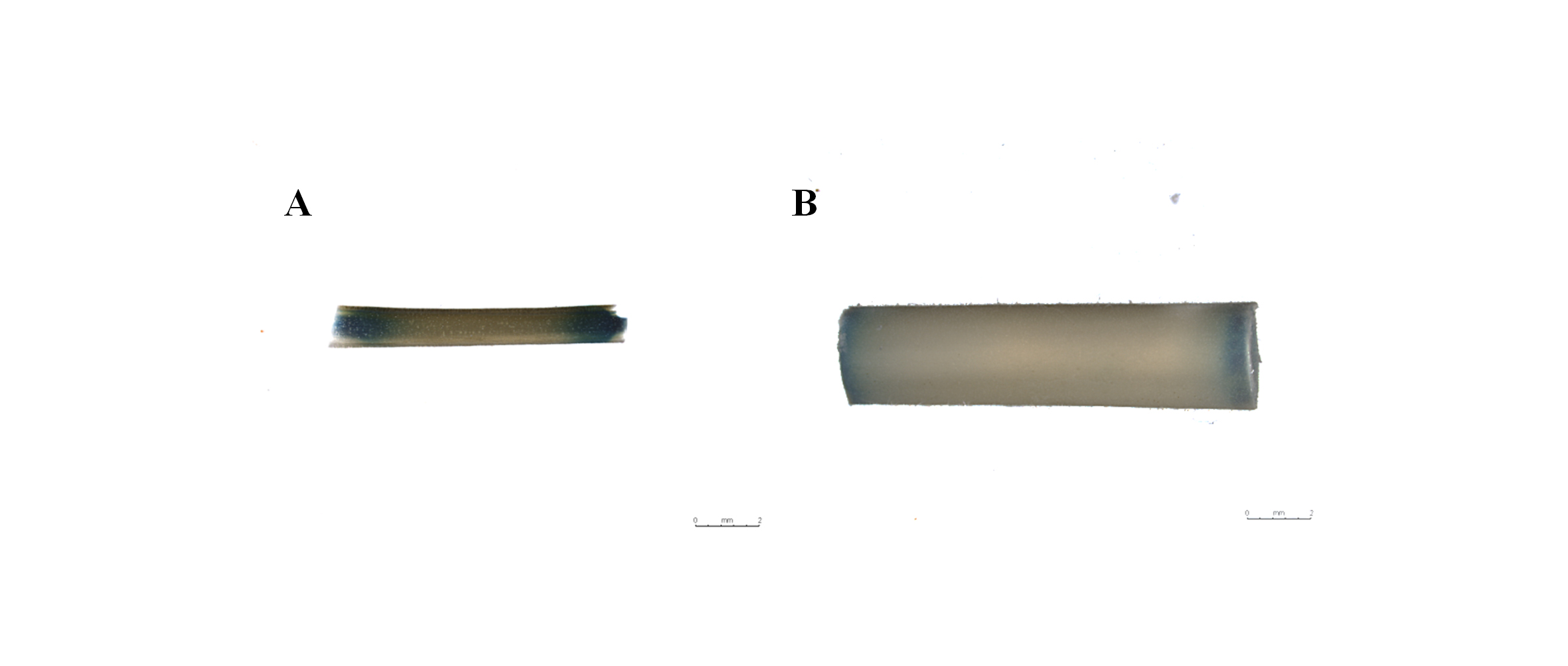

Supplement: Figure S3 — Histochemical GUS staining of stems and leaf pedicels of SlDOF10pro::GUS transgenic tomato plants. (A) Leaf pedicel and (B) stem. Scales bars are: 2 mm. [file Image_3.JPEG]

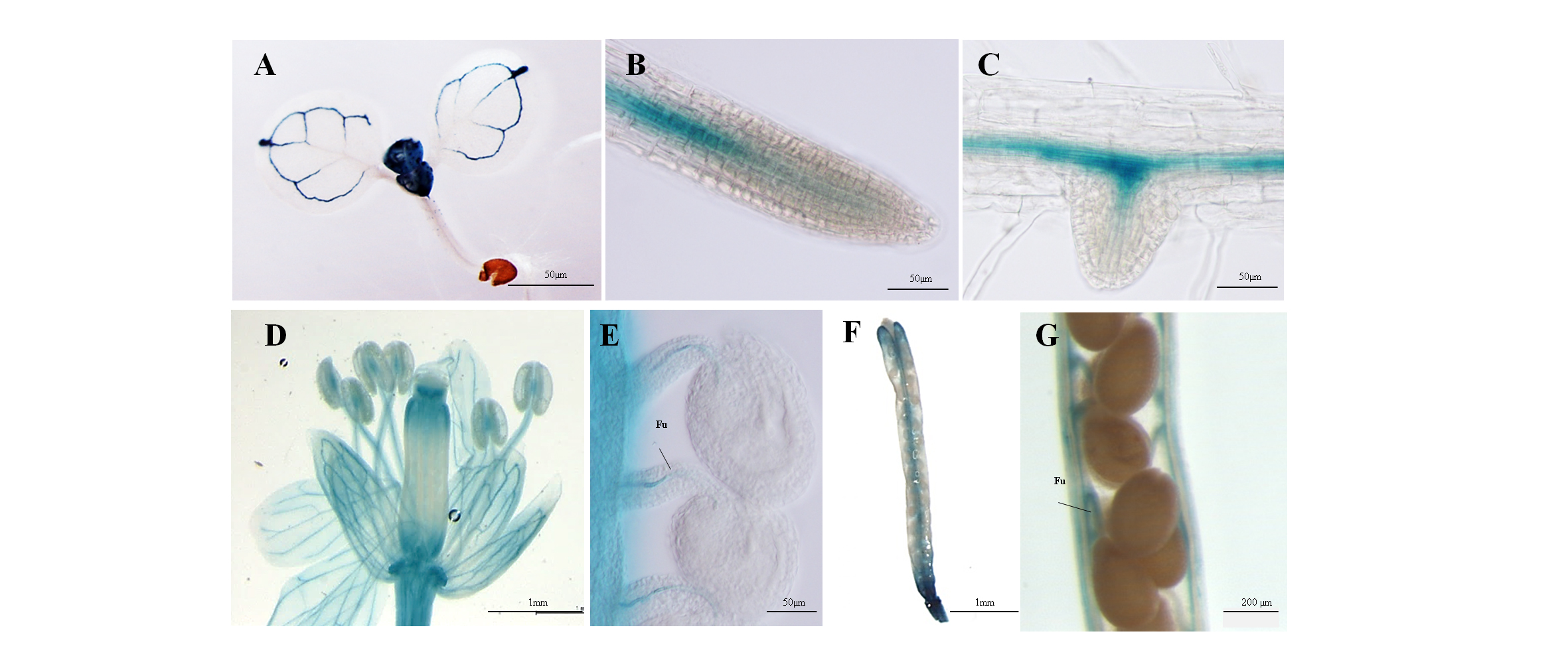

Supplement: Figure S4 — Histochemical GUS staining of transgenic Arabidopsis carrying the GUS-coding region fused to SlDOF10 promoter. SlDOF10pro::GUS expression in the vascular tissue of seedlings (A), roots (B), secondary roots (C), and flowers (D). Dissected flower showing GUS staining at the vascular tissue from the funiculus (E). Mature fruit showing GUS staining in the apical and basal region and the margin of the valve (F). Detail from mature fruit showing blue staining in the funiculus (Fu) and vascular tissue from the fruit (G). Scale bars are: 1 mm. [file Image_4.JPEG]

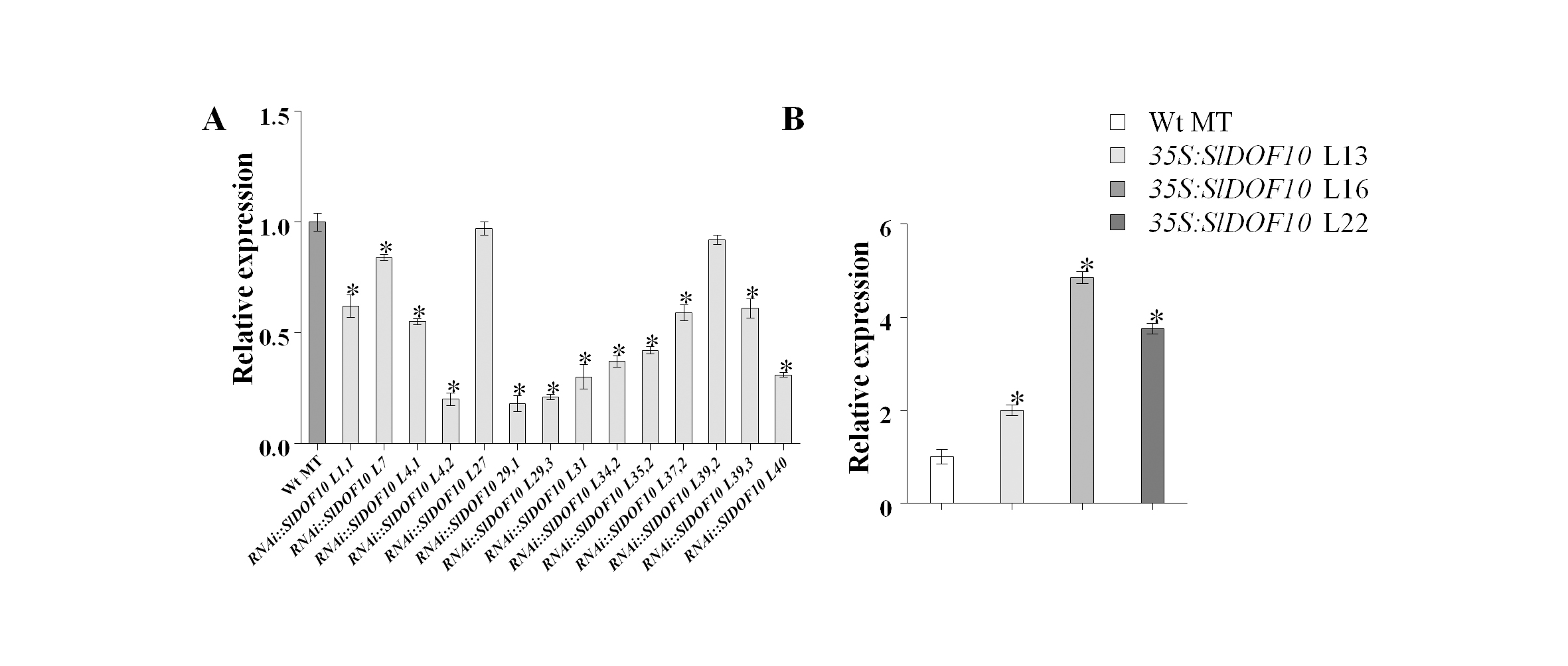

Supplement: Figure S5 — Expression level of SlDOF10 gene in the RNAi and overexpressing lines. (A) Relative expression of SlDOF10 in the SlDOF10-RNAi lines measured by qRT-PCR. (B) Relative expression of SlDOF10 in the 355S:RNAi lines measured by qRT-PCR. The asterisks denote a significant difference between the transgenic lines and the wild type at p < 0.05. [file Image_5.JPEG]

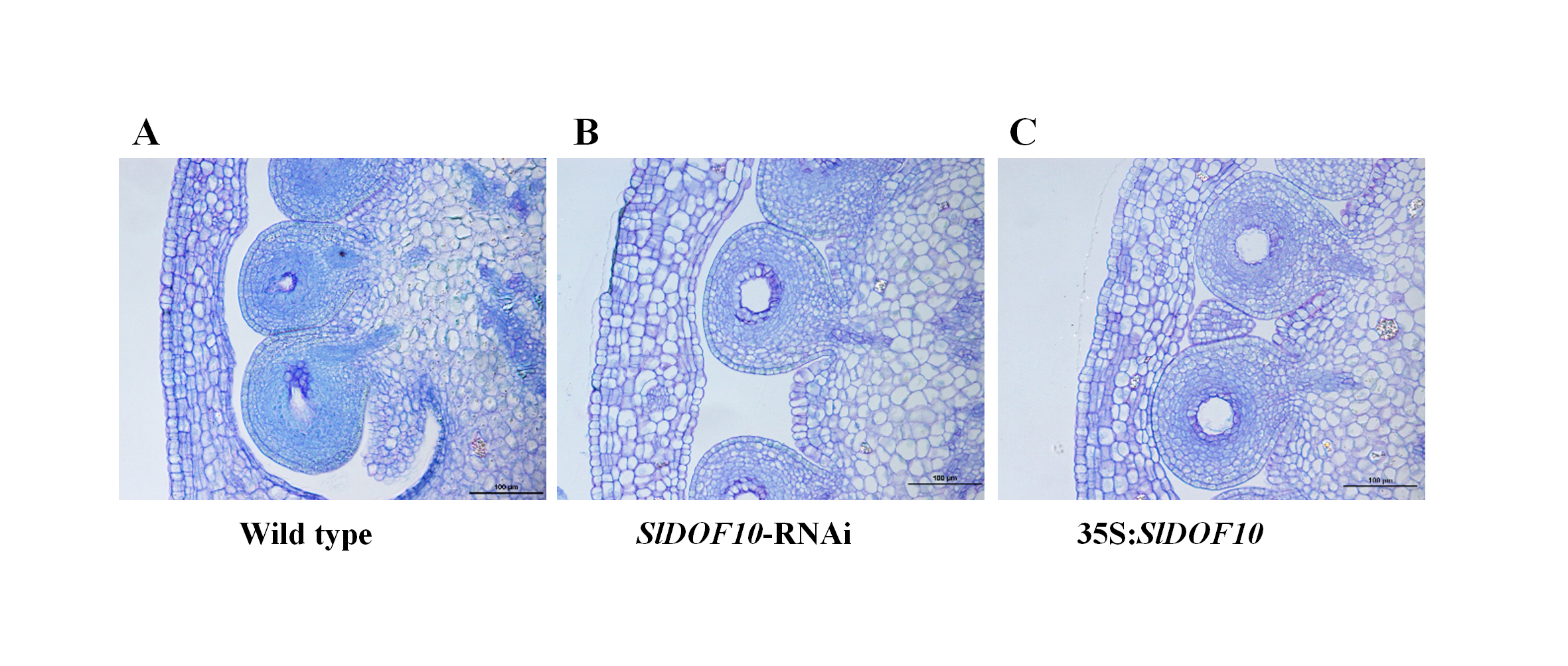

Supplement: Figure S6 — Histological sections of ovules from flowers in anthesis. (A) Wild type ovules (Micro-Tom cv.). (B) SlDOF10-RNAi plants. (C) 35S: SlDOF10 plants. [file Image_6.JPEG]
